# Small RNA Changes in Plasma Have Potential for Early Diagnosis of Alzheimer’s Disease before Symptom Onset

**DOI:** 10.3390/cells13030207

**Published:** 2024-01-23

**Authors:** Joanna Palade, Eric Alsop, Amanda Courtright-Lim, Michael Hsieh, Timothy G. Whitsett, Douglas Galasko, Kendall Van Keuren-Jensen

**Affiliations:** 1 Neurogenomics Division, Translational Genomics Research Institute (TGen), Phoenix, AZ 85004, USA; jpalade@tgen.org (J.P.); ealsop@tgen.org (E.A.); mcfhsieh@gmail.com (M.H.); twhitsett@tgen.org (T.G.W.); 2 Biomedical Ethics Program, Mayo Clinic, Scottsdale, AZ 85259, USA; acourtright.lim15@gmail.com; 3 Department of Neurosciences, San Diego and Shiley-Marcos Alzheimer’s Disease Research Center, University of California, La Jolla, CA 92037, USA; dgalasko@health.ucsd.edu

**Keywords:** small RNA sequencing, Alzheimer’s disease, mild cognitive impairment, biomarker, plasma, extracellular vesicle

## Abstract

Alzheimer’s disease (AD), due to its multifactorial nature and complex etiology, poses challenges for research, diagnosis, and treatment, and impacts millions worldwide. To address the need for minimally invasive, repeatable measures that aid in AD diagnosis and progression monitoring, studies leveraging RNAs associated with extracellular vesicles (EVs) in human biofluids have revealed AD-associated changes. However, the validation of AD biomarkers has suffered from the collection of samples from differing points in the disease time course or a lack of confirmed AD diagnoses. Here, we integrate clinical diagnosis and postmortem pathology data to form more accurate experimental groups and use small RNA sequencing to show that EVs from plasma can serve as a potential source of RNAs that reflect disease-related changes. Importantly, we demonstrated that these changes are identifiable in the EVs of preclinical patients, years before symptom manifestation, and that machine learning models based on differentially expressed RNAs can help predict disease conversion or progression. This research offers critical insight into early disease biomarkers and underscores the significance of accounting for disease progression and pathology in human AD studies.

## 1. Introduction

Over 50 million people worldwide currently live with Alzheimer’s disease (AD), a number predicted to double in each coming decade [1], causing significant economic and emotional burdens to the affected and their families. AD is a multi-factorial neurodegenerative condition characterized by progressive neuronal loss and neuroinflammation associated with amyloid plaque (Aß) deposition and neurofibrillary tangles (NFTs). While disease progression varies widely between individuals, it generally entails a preclinical stage, where neurological changes are present without evident symptoms, followed by mild cognitive impairment (MCI) with slight neurological decline, finally culminating in AD, which encompasses both distinct brain pathology and prominent cognitive symptoms [2]. The NIA-AA has created pathology-driven guidelines for research [3], which have helped simplify patient categorization based on pathology (postmortem confirmation or in vivo biomarker changes) and not on clinical symptoms. However, as new disease-modifying agents become available, it will be beneficial to identify biomarkers that correlate with symptoms as well as pathology.

PET imaging is used to evaluate Aß deposition and MRI can be used to assess brain atrophy that occurs progressively in AD patients. Cerebrospinal fluid (CSF)-based tests measuring NFTs and the ratio of Aß1–40 and 1–42 can identify changes that predate disease by a decade [4,5]. However, the practical implementation of brain imaging and CSF sampling in the clinic, to non-symptomatic subjects, is restricted by access and a reluctance to provide an invasive CSF sample. Additionally, these tests become less precise in aged patients. The prevalence of positive PET imaging in cognitively unimpaired adults increases with age, and 20% of PET-positive individuals at age 70 have no clinical symptoms [6].

As disease-modifying interventions have the greatest potential benefit during the early stages of AD [7], the development of accessible, minimally invasive, and cost-effective biomarker tools is a critical unmet need. Over the past decade, numerous groups have investigated disease-associated changes in extracellular vesicle-associated small RNA (exRNA) from accessible biofluids, like plasma or serum. As a result, many differentially expressed miRNA transcripts have been described. The miR-29 family, known to target genes associated with neurological function, was detected in blood and CSF [8,9,10,11]. miR-93 levels were lower in the serum of AD patients and correlated with pathology severity, as classified via Braak staging in postmortem samples [12,13]. Gender and APOE genotype were found to influence the expression of miRNAs in CSF EVs in control and AD patients [14]. Members of the miR-let-7 family, miR-17, -143, -151, -23, and -191, and numerous other transcripts have been implicated in neurodegeneration in various studies [8,11,12,13,15,16,17].

While there has been a focus on miRNA in particular, other small RNA biotypes may represent viable biomarker candidates as well. Small fragments of YRNAs are particularly abundant in plasma and tRNA transcript expression may be associated with stress, although the function and biology of these small RNA subspecies are poorly understood [18,19,20,21,22,23,24].

Cell-free RNA in circulation is typically encapsulated in or associated with extracellular vesicles (EVs) or bound within non-vesicular nanoparticles [25,26], circulating protein complexes, exomeres, ribonucleoproteins (RNPs), HDL, and LDL, all of which may protect exRNAs from degradation or facilitate transport.

EVs are membrane-enclosed, nanosized particles of cellular origins. Since EVs are found in all human biofluids and carry cell-derived protein and nucleic acid cargo, they represent an accessible, minimally invasive source of biomarkers with predictive, prognostic, and diagnostic potential [27,28,29,30].

However, non-vesicular nanoparticles have also been found to have diagnostic capabilities, particularly in the context of cancer and coronary disease [31,32]. In addition, some RNP and HDL/LDL carriers have long half-lives in circulation, providing unique biomarkers with potentially more stability [33,34]. Therefore, we wanted to evaluate whether or not these other nanoparticle species provided either a less variable signal or a potentially unique one for monitoring chronic disease.

Here, we evaluated EV-associated RNA vs. EV-depleted RNA as potential biomarkers based on RNA diversity and variability. We then described differences in the plasma small RNA EV cargo in a cohort of AD, MCI, and non-cognitively impaired control patients. We used these data to construct models for predicting disease. We identified transcriptomic changes in EV-RNAs that precede formal diagnosis and symptom onset in a small subset of the samples.

## 2. Materials and Methods

### 2.1. Sample Collection

The samples were collected at the University of California San Diego Alzheimer’s Disease Research Center from 2000–2011. This study was approved by the University of California, San Diego Human Research Protections Program, protocol 171823. Patient recruitment, cognitive assessment, diagnosis, monitoring, and treatment were carried out in association with the University of California San Diego Alzheimer’s Disease Research Center by trained medical personnel. Along with other clinical and neurocognitive assessments, the Dementia Rating Scale (DRS) and Mini-Mental State Exam (MMSE) were administered. When patients were seen at the clinic for assessment and blood draw, they could also be consented to future autopsy. Since the initial enrollment, several of the individuals in this study have come to autopsy, providing important details regarding the accuracy of clinical diagnoses. The guidelines and criteria described by Salmon et al. were adhered to for postmortem neuropathological evaluation [31]. All patients or their respective surrogates provided written informed consent for participation in this study. All aggregated sequencing data used here, including counts for individual RNAs, can be found in the ERCC extracellular RNA atlas, accession EXR-KJENS1yrpQwk-AN (https://exrna-atlas.org/exat/datasets/) or in SRA, accession PRJNA972172 ID: 972172, release date: 26 July 2023. Blood was collected in EDTA tubes; the supernatant (plasma) was collected and stored in 1 mL aliquots at −80 °C until thawed for RNA isolation and sequencing.

### 2.2. RNA Isolation

Plasma EV RNA was isolated using QIAGEN’s ExoRNeasy Midi kit according to the manufacturer’s instructions. The 1 mL plasma aliquots were mixed with binding buffer and applied to the ExoRNeasy filter column to capture EVs. The EV-depleted flow-through was retrieved and stored separately. The EVs were washed and lysed with QIAzol. After a phenol-chloroform extraction and ethanol precipitation, the RNA was applied to a MinElute column, washed, and eluted with ultrapure RNase-free DNase-free water. For the EV-depleted fraction, mirVana PARIS RNA isolation (ThermoFisher, Waltham, MA, USA) was used. Briefly, the cell-free RNA was isolated via phase extraction with acid phenol-chloroform, precipitated with ethanol, applied to the column, and washed. After elution with 95 °C water, the RNA underwent DNase treatment (TURBO DNA-free Kit for DNA removal, Invitrogen, Waltham, MA, USA) according to the manufacturer’s instructions. Finally, RNA was cleaned and concentrated using Zymo’s RNA Clean & Concentrator-5 and eluted with ultra-pure water. Concentrations for all cell-free RNA were determined with Quant-iT RiboGreen (ThermoFisher, R11490), using the low-range standard curve and an Agilent Cytation plate reader.

### 2.3. Small RNA Library Preparation and Sequencing

Libraries were generated with Perkin Elmer’s NEXTFLEX Small RNA-seq v2 kit. Cell-free RNA from the 1 mL plasma samples and 3′ adenylated adapters were denatured at 70 °C and underwent 3′ ligation for 2 h at 22 °C. Agencourt AMPure XP Magnetic Beads were used to clean up the RNA, followed by 5′ ligation and cDNA synthesis. PCR was carried out with NEXTflex Barcode Primers for 18 cycles, and libraries were bead-cleaned and size-selected using 6% polyacrylamide gel electrophoresis, according to the manufacturer’s instructions. Library size and concentration were determined via Agilent 2100 Bioanalyzer, using the High Sensitivity DNA kit. For sequencing, libraries were pooled equimolarly, and the pools were assayed with the Agilent High Sensitivity DNA chip. The pools were denatured with NaOH, spiked with 5% PhiX, and clustered onto Illumina V3 flow cells. Sequencing was carried out on Illumina’s HiSeq 2500 using TruSeq v3 reagents, for 50 cycles, with a 7-cycle indexing read.

### 2.4. RNA-Sequencing Data Analysis

The raw sequence image files from the Illumina HiSeq (bcl files) were converted to the fastq format using bcltofastq v. 2.19.1.403 and checked for quality to ensure the quality scores did not deteriorate at the read ends. All samples were processed using the exceRpt small RNA pipeline [32] and by-sequence analysis was performed as described previously [33]. Briefly, reads in fastq files were trimmed to remove TruSeq adapters from the 3′ end along with random 4 bp primers added by the NEXTFLEX v2 kit to the 3′ and 5′ ends using cutadapt. Reads shorter than 15 nucleotides after adapter trimming were discarded. After trimming, the count tables of sequences for each sample were generated by first comparing the adapter trimmed reads against the human genome mapped BAM files from exceRpt to find reads that mapped to the human genome. The genome-mapped reads were then identified in the transcriptome-mapped BAM file to identify the biotypes associated with each read. Since multi-mappers are common with small RNA (short read lengths), biotype was assigned in the priority order used in the exceRpt pipeline: miRNA, YRNA, tRNA, piRNA, and protein-coding biotypes, followed by a bin containing any other annotation in Ensembl (pseudogenes, lncRNAs, etc.). Reads with the same sequence (after adapter trimming) were then collapsed into a counts table with biotype and gene annotations. Genes from miRbase (miRNAs), piRNABank (piRNAs), and GtRNAdb (tRNAs) were annotated using the IDs from their respective databases. Protein coding genes and yRNAs from GENCODE are reported as their gene names. All other genes in GENCODE (antisense, lincRNAs, misc RNAs, pseudogenes, mitochondrial RNAs, etc.) are reported using their GENCODE transcript IDs. New gene IDs for all gene isoforms were generated by concatenating a number to the gene ID and a look-up table was generated that contains the new gene IDs and the actual gene sequences (Supplementary Materials Table S2). Isoforms were kept as separate transcripts if they differed by one or more nucleotides. All count tables (both genes and sequences) were then loaded into R (v. 4.2.2) for further analysis. Samples that did not contain more than one million reads mapped to a transcriptomic feature were excluded from all analyses.

### 2.5. Differential Expression Analysis

Differential expression was conducted using the DESeq2 package (version 1.38.3) in R for all sequences that had expression levels > 10 read counts in at least 50% of samples, in each comparison. The raw read counts for the samples were normalized using the median ratio method (default in DESeq2). The Benjamini–Hochberg method was used for reported adjusted *p*-values. Complete tables of differential expression analysis results can be found in the Supplementary Materials Table S4.

### 2.6. Classification Analysis via Elastic Net

Elastic net analysis was performed using the median ratio normalized counts for all sequences from DESeq2, as described in Kalani et al. [34], using Elastic Net instead of LASSO to reduce potential overfitting by a LASSO model. Following DESeq2 differential expression analysis, sequences with unadjusted *p*-values < 0.05 between sample groups (see above) were selected to be input into the classification model. Modeling was conducted using the glmnet package (version 4.1.7) in R. A series of loops were implemented to (1) identify the optimal lamda value for an elastic net model, (2) select sequences that produced the best performing elastic net model, and (3) estimate performance metrics (accuracy and AUC) for the best-performing model. In the first loop, we performed a series of 1000 iterations of cv.glmnet using random 80/20 training/test set splits. For each iteration, the predict() function was used to calculate the accuracy for predicting the test set, and the lambda.min that corresponded to the iteration with the highest accuracy was selected as the optimal lambda value for our model. In the second loop, we performed a series of 1000 iterations of glmnet using random 80/20 training/test set splits, but with a constant lambda (selected in step 1), to determine which sequences resulted in the best elastic net model (the sequences found to be most important in the iterations with the best predictive value). The iteration with the highest prediction accuracy was called the best elastic net model and the sequences corresponding to this model were selected and reported as the sequences used for the elastic net classifier. To demonstrate the best model accuracy and generate waterfall plots and heat maps, the best model was tested using all samples and the ability of the model to accurately place each sample was reported using the glmnet predict() function. In the third and the last loop, we performed a final series of 1000 iterations of glmnet using random 80/20 training/test set splits but kept the lambda and feature set constant (as determined in steps 1 and 2 above). This allowed a more robust average accuracy and AUC to be calculated using randomized training and testing sets from the reported best model. AUCs were determined from cv.glmnet() using type.measure = “auc” using the best elastic net model. Converters were then added to the best model generated using the non-converter samples and the predict() function was re-run with this data set containing converter and non-converter samples to see where the model placed the converter samples. Waterfall plots and heatmaps containing converters were then generated based on the outcome from the predict() function.

### 2.7. Classification Analysis via Linear Regression Modeling

Linear regression modeling (LRM) was performed using the MASS R package. In R, the glm (generalized linear model) approach was used with a data frame containing the gene counts for the top 10 differentially expressed genes (by adjusted *p*-value or abs(log2FC)) and a response variable representing each sample’s group (Supplementary Materials Table S6). Testing was carried out using a binomial distribution as all comparisons were between two groups using the predict.glm() function. ROC curves from the LRM model were calculated using the pROC package and roc() function, and an AUC value for the ROC curve was calculated using the auc() function. Average and standard deviation values were calculated from both model accuracy and AUC by re-running the modeling 100 times with randomly selected 80/20 training and testing sample subsets.

## 3. Results

### 3.1. EV-Associated RNA Cargo Is More Diverse and Less Variable Than EV-Depleted RNAs in Plasma

This cohort was composed of AD and MCI patients and an age-matched control group (defined as having no diagnosed neurodegenerative conditions at the time of plasma collection), with a balanced male (*n* = 65): female (*n* = 67) ratio. Scores for the Dementia Rating Scale (DRS) and the Mini-Mental State Examination (MMSE) were available and reflected the cognitive status of all participants (Supplementary Materials Table S1). The DRS is made up of 24 tasks that generate scores for Attention, Construction, Conception, Memory, and Initiation/Perseveration. In previous studies [35], a cutoff of 136 separated healthy controls from AD and MCI and MCI and AD could be separated at 124. For the MMSE, the scores range from 0–30, with lower scores being the most severe. Mild stages of the disease score 20–26, moderate is 10–19, and severe is <10 [36].

We chose a commercial EV isolation method suitable for processing large numbers of samples (*n* = 132) with high reproducibility [37,38]. This method also enabled the straightforward capture of EV-depleted biofluid, allowing for further analysis of exRNAs not found within EVs, such as RNAs associated with RNA-binding proteins, HDL, LDL, etc.

**Figure 1:**
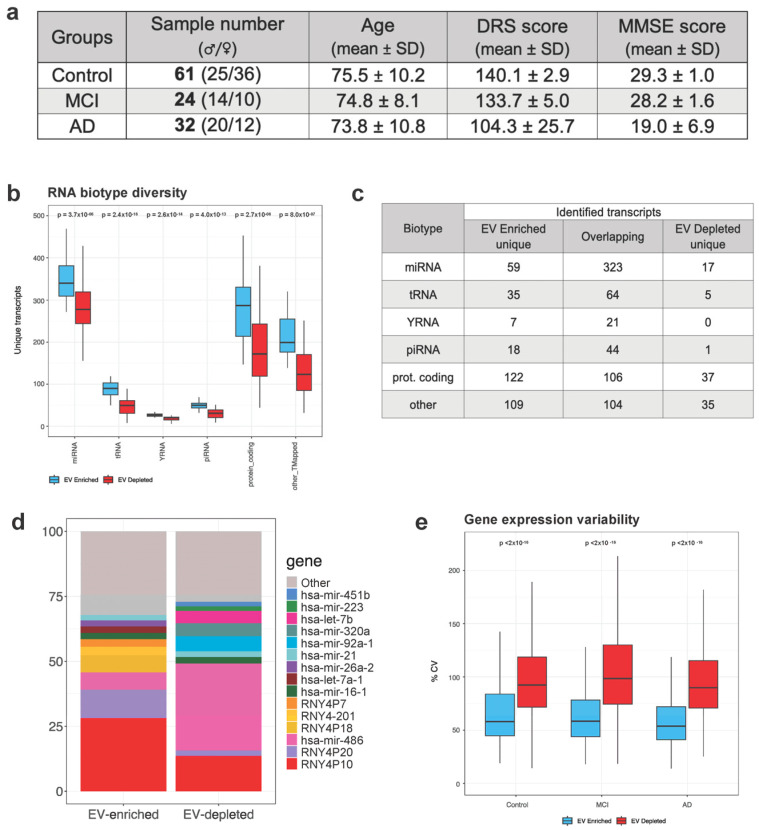
Plasma EVs are a rich source of small RNA compared with the EV−depleted fraction. (**a**) 117 EV samples passed quality sequencing metrics and were used for this study. Age ranges were evenly distributed among the experimental groups. Mean cognitive test scores decreased with disease severity. (**b**) For 86 samples, the small RNA was also sequenced from the EV-depleted fraction, and 46 samples fulfilled sequencing quality criteria. miRNA, tRNA, YRNA, piRNA, and protein-coding biotypes were identified in both isolations. The EV-enriched portion consistently had greater transcript diversity (unique RNAs). (**c**) The majority of transcripts were found in both EV-enriched and EV-depleted samples, with the highest number of unique transcripts found in EVs. (**d**) A display of the 10 most abundant transcripts in each isolation method, EV-enriched or EV-depleted, shows enrichment for YRNA4P10 in EVs and miR-486 in EV-depleted samples. (**e**) EV-enriched RNA had lower variability compared with the EV-depleted RNA, as assessed by the % of CVs of all genes with normalized expression levels > 10 counts in >50% of the samples’ most expressed genes, regardless of disease status. Statistical analysis (**b**,**e**) was carried out via *t*-test.

Plasma exRNA exists within EVs or is associated with non-vesicular protein complexes. To compare the diversity and consistency of each type, we prepared libraries and sequenced the small RNA contents from the EV-enriched and EV-depleted fractions. Samples with less than 1,000,000 reads mapping to the transcriptome were excluded from the analysis (Figure 1a). This quality filtering step eliminated 11% of the EV-enriched samples and 47% of the EV-depleted samples (leaving *n* = 117 and *n* = 46 for the EV-enriched and EV-depleted fractions, respectively, that fulfilled the quality criteria). RNA was aligned using exceRpt [32], allowing us to measure the abundance of different RNA biotypes (miRNA, tRNA, yRNA, piRNA, and protein-coding biotypes). All small RNA species were identified in both the EV-enriched and EV-depleted samples; however, EV-enriched samples had significantly greater RNA transcript diversity (Figure 1b). Few sequences were exclusively identified in the EV-depleted fraction, whereas numerous transcripts were unique to EVs. A total of 15% of the total detected miRNA and 34% of the total detected tRNA transcripts were found in EVs only (Figure 1c). A total of 60% of all sequences were identified in both sample types, although the relative abundance of transcripts differed. RBC-associated miR-486 makes up over a third of all transcriptome reads in the EV-depleted fraction but only 7% of the EV reads. YRNA, on the other hand, is more abundant in EVs compared with EV-depleted samples (54% vs. 19%) (Figure 1d). Several CNS-tissue-enriched miRNAs (miR-5586, -346, -770, and -383) are found in EVs exclusively [33]. Interestingly, the liver-enriched miRNA miR-122b [39] is found primarily in the EV-depleted fraction, where it makes up over 1% of total reads, and the pancreas-enriched miRNA miR-216a-5p [40] is detected in the EV-depleted fraction only (Supplementary Materials Table S3). The EV-enriched and EV-depleted RNA from matched samples are well characterized and may provide a useful tool for better understanding cell-free RNA distribution within human plasma (exRNAs found in matched EV and EV-depleted samples can be found in Supplementary Materials Table S3). A coefficient of variation analysis (%CV) of all genes with normalized expression levels >10 counts in >50% of samples between both groups showed significantly less variability in the EV-enriched fraction, regardless of disease status (Figure 1e, *p* < 2 × 10^−16^, *t*-test). Overall, EV-RNA samples demonstrated increased small RNA transcript diversity and a greater abundance of unique sequences and were more likely to fulfill the transcriptome mapping requirements (>1,000,000 mapped reads). Taken together, these findings supported the use of EV-enriched exRNAs for biomarker discovery in our plasma samples, and we chose to focus on the EV cargo for the remainder of our analysis.

### 3.2. Postmortem Pathology Does Not Always Match Clinical Diagnosis and May Present a Confounding Variable for Gene Expression Analysis

**Figure 2:**
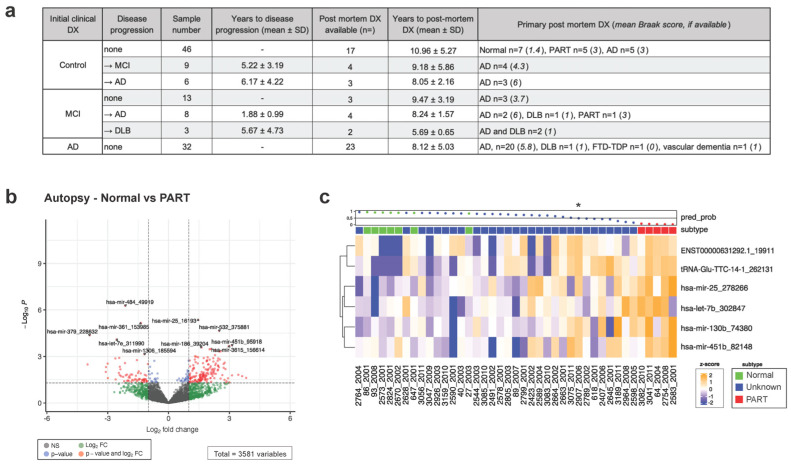
Postmortem diagnosis and disease progression information complicates the initial cohort sample grouping. (**a**) Autopsy findings for a fraction of our cohort show that initial diagnosis does not always correspond with postmortem brain pathology and that several individuals from the control and MCI groups progressed in disease status after sample collection. (**b**) Gene expression comparison of controls with confirmed normal vs. PART pathology is depicted as a volcano plot, where red dots represent sequences that are log(2)fc > 1 and a *p*-value < 0.05. (**c**) Samples with unknown postmortem diagnosis from the control group (blue) were analyzed using the pathology-based model (asterisk denotes the 0.5 ELNET model probability cutoff used for sample inclusion criteria). The waterfall plot across the top of the heatmap demonstrates ELNET model confidence in assigning groups (0 to 1, with 0.5 being no confidence in the assignment), with dots colored to the diagnosis group, while the sequences picked by ELNET are displayed on the right of the heatmap. The naming scheme for RNA biotypes is described in Section 2 (RNASeq data analysis). PART = primary age-related tauopathy, DLBs = dementia with Lewy bodies, FTD = frontotemporal dementia, and DX = diagnosis.

AD and other related dementias, such as frontotemporal dementia (FTD-TDP), vascular dementia, and dementia with Lewy bodies (DLBs) can be misdiagnosed, as they may manifest overlapping symptoms early in the disease course, and amyloid deposition increases with age [41,42,43,44]. All of the blood draws for this study occurred at one of the patient visits to the AD clinic. Since the time of the blood draws, several of the participants came to autopsy and had neuropathological evaluations that could be used for verification of diagnosis. A formal diagnosis is only possible postmortem upon examination of the brain. Exploring this cohort’s metadata revealed that postmortem findings did not always match the initial diagnosis (Dx) (Figure 2a). While a subset of the control group was found to have normal age-related pathology (*n* = 7), five individuals had more significant tau pathology (in the absence of amyloid plaques) and scored as having Primary Age-Related Tauopathy (PART) at autopsy [45,46], potentially introducing a confounding variable for analysis. We therefore examined differential gene expression between the control individuals with known postmortem diagnosis (normal vs. PART, *n* = 12, autopsy 8.8 ± 3.2 years post-sample collection). To increase the number of potential targets and due to the nature of the NEXTFLEX v2 library preparation kit capturing the entire small RNA, we did not collapse to canonical gene IDs but instead performed the analysis using all unique sequences, even when they differed by a single nucleotide. A table linking each gene ID used in this study with its sequence, canonical gene ID, and biotype is found in the Supplementary Materials Table S2. Using our expanded sequence-based feature list, we analyzed the small RNA gene expression in plasma samples and found 266 differentially expressed (DE) transcripts in patients with subsequent confirmed normal age-related pathology compared with confirmed PART pathology (*p* < 0.05, Figure 2b). Among the DE transcripts were miRs -224, -22, and -484, which have known neurological involvement [47,48,49,50]. As postmortem Dx was not available for the rest of our control cohort (*n* = 29), we constructed a predictive model using Elastic Net (ELNET). The ELNET model was built using controls with autopsy data split into controls with normal age-related pathology and controls with PART pathology. The best ELNET model was able to distinguish these groups with 100% accuracy using six sequences (Supplementary Materials, Figure S1a and Table S5). Inserting the control individuals without confirmed autopsy data into the model identified eight samples as potentially having PART, with 50% or greater confidence, according to the model (from the asterisk to the right in Figure 2c). In order to increase our confidence that the control group had minimal pathology, the subjects with signatures resembling PART were excluded from further analysis (Figure 2c). Additionally, five control participants had AD pathology upon postmortem examination (which took place 4–21 years post-diagnosis) and were also excluded (Supplementary Materials, Table S1 and Figure S1).

At autopsy, a number of individuals from our MCI and AD groups had low Braak scores and primary pathology associated with PART, DLBs, FTD-TDP, and vascular dementia instead (Figure 2a). A subset of the MCI group was diagnosed with DLBs post-sample collection (*n* = 3, Figure 2a). To avoid confounding variables, these participants were excluded from subsequent analysis.

Finally, a subset of our control and MCI cohort progressed in their clinical diagnosis after sample collection (referred to here as “converters”; Figure 2a). These converters provide us with a valuable opportunity to investigate the molecular signature that indicates future progression and precedes symptom onset/increased severity. The samples from converters were kept as a separate group for analysis. Integration of postmortem diagnosis and conversion status resulted in redefined experimental groups with more definitive pathology and disease trajectory (Supplementary Materials Figure S1b).

### 3.3. MCI and AD Have Distinct RNA EV Signatures Comprised of Differentially Expressed miRNA, tRNA, YRNA, and piRNA Species

**Figure 3:**
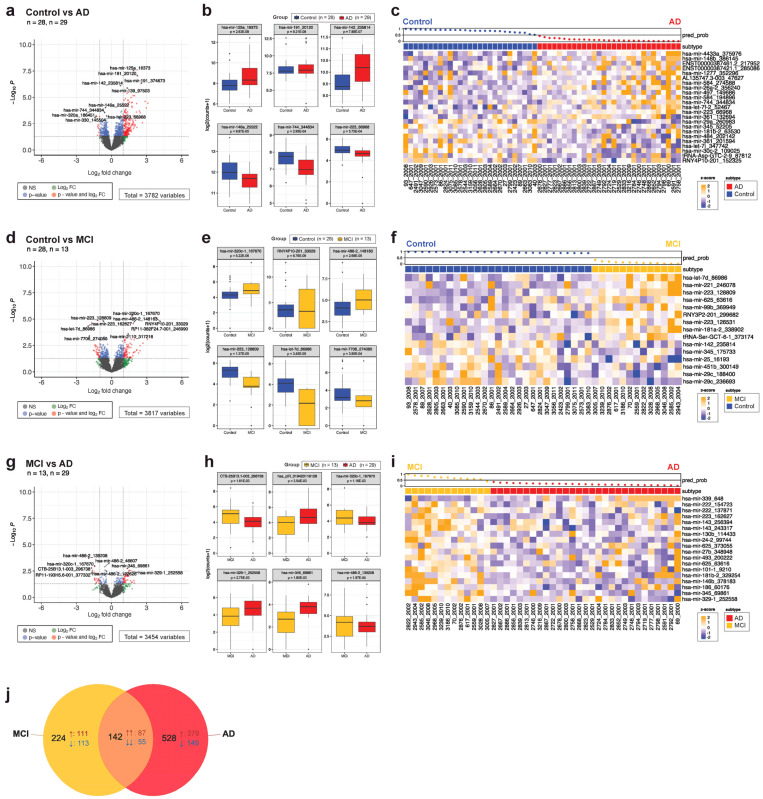
Differentially expressed small RNA transcripts in MCI and AD are both distinct and overlapping. Differential gene expression analysis is shown as volcano plots between control and AD (**a**), control and MCI (**d**), and MCI and AD (**g**) groups. Box plots of the top 3 most significantly upregulated and downregulated transcripts are shown for each comparison: control vs. AD (**b**), control vs. MCI (**e**), and MCI vs. AD (**h**). ELNET was used to select features from the DE gene list to construct a classification algorithm for each comparison: control vs. AD (**c**), control vs. MCI (**f**), and MCI vs. AD (**i**). (**j**) A Venn diagram of the DE sequences between AD and MCI displays the overlapping transcripts, all of which are concordant. ↑ arrows denote transcripts that are upregulated relative to the control group, while ↓ arrows mark downregulated transcripts. The *p*-value (unadjusted) for each comparison is displayed for each box plot (**b**,**e**,**h**). The waterfall plot across the top of the heatmap demonstrates ELNET model confidence in assigning groups (0 to 1, with 0.5 being no confidence in the assignment), with dots colored to the diagnosis group.

To investigate how the EV small RNA cargo changes with disease, we first carried out a DE analysis between the groups that retained the same clinical diagnosis throughout the time of the study. Differential expression analysis between the control and AD groups yielded 670 sequences that were significantly different (Figure 3a, *p* < 0.05). DE sequences from any RNA biotype or isoform of an annotated transcript were included. Over 30% of the DE signature was made up of sequences that did not come from miRNA but came instead from YRNA, tRNA, piRNA, and protein-coding transcripts (Supplementary Materials Table S4). Multiple sequences (isomiRs, sequences that differ by >1 nucleotide from the annotated sequence) of miR-125a, -139, -142, -191, and -23a are consistently and significantly upregulated in our AD data set, whereas isomiRs of miR-744 and -223 are downregulated in disease (Figure 3b).

MCI can precede AD and presents with a subtle decline in cognition. We examined the small RNA expression levels between the control and MCI groups and found 366 significant transcripts, 80% of which were made up of miRNAs (Figure 3d). miR-320c, previously identified in plasma from early AD subjects [51], CSF, and saliva in relation to traumatic head injuries [52,53] and in brain tissue after stroke [54], is upregulated in the MCI cohort (Figure 3e).

To examine whether the MCI transcriptional signature is distinct from the AD signature, or if RNA sequences change significantly with disease progression, we examined differential gene expression between those groups and found 132 significant transcripts (Figure 3g). Transcripts of miR-320c, -345, -26a are differentially expressed between the two groups (Figure 3h). Indeed, when comparing the DE transcripts in AD (relative to control) and MCI (relative to control), overlap is restricted to 142 sequences (Figure 3j). Several miR-191 isomiRs, for example, were significantly upregulated in AD only, whereas miR-320c upregulation was restricted to MCI. Notably, the overlapping transcripts are all concordant, suggesting a partially shared disease signature (Figure 3j).

For clinical utility, biomarkers must distinguish the disease from the control and closely related conditions with high sensitivity and specificity. We compared two approaches, ELNET and logistic regression modeling (LRM), to assess the value of our differential expression analysis in predicting AD and MCI. LRM entails the manual selection of genes based on *p*-value and fold change, unlike ELNET, where the genes included in the model are selected by a machine learning algorithm. Here, we used the top 10 most significantly different sequences for each comparison, either by (1) *p*-value or (2) highest absolute fold change (Supplementary Materials Table S4). ELNET could distinguish between the control, MCI, and AD with 78–83% accuracy, using predominantly miRNA transcripts (Figure 3c,f,i, Supplementary Materials Table S5). LRM performed worse across all comparisons, with 52–65% accuracy (Supplementary Materials Table S6). There was no overlap between the transcripts chosen by ELNET to construct a model and the 10 sequences with the lowest *p*-value/highest fold change used for LRM.

### 3.4. Cognitively Unimpaired Controls That Later Developed MCI or AD Display a Disease-Associated EV-RNA Signature Predating Symptom Onset

**Figure 4:**
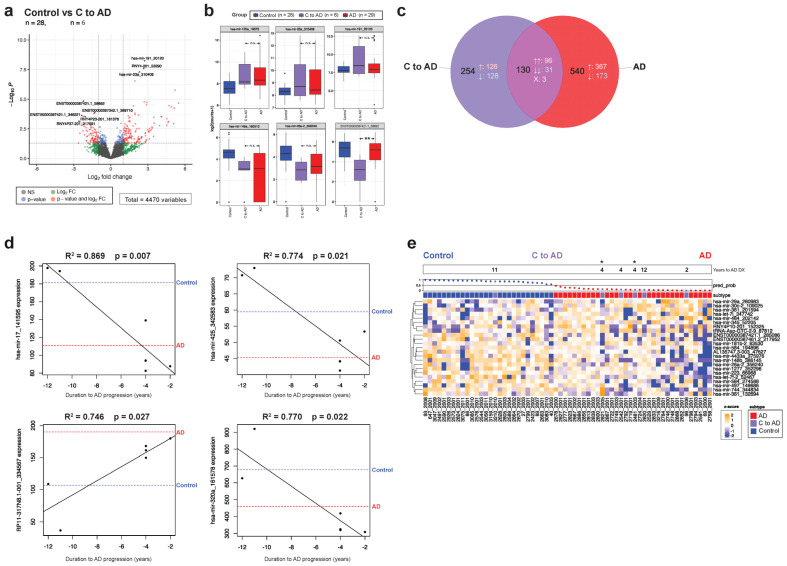
Changes in EV-RNA predate symptom onset in the preclinical AD cohort. (**a**) Differential gene expression analysis between the control non-converters and control pre-AD (C to AD) found 384 significant transcripts. (**b**) Representative transcripts that are significantly up/downregulated in the C to AD group compared with non-converter controls are depicted as box plots, with expression levels in control, C to AD, and AD shown. (**c**) A third of DE transcripts in the C to AD group are concordant and overlapping with AD DE transcripts. (**d**) Regression analysis by time to disease conversion with corresponding R^2^ and *p*-values are displayed, where the *X* axis marks the years to approaching disease diagnosis (e.g., -4 denotes a participant diagnosed with AD 4 years after the blood draw); the median expression level for the transcript in control non-converters (blue) and AD (red) is provided as a reference. (**e**) The ELNET control vs. AD model is used to determine whether the C to AD samples can be accurately predicted, with the top bar marking years elapsed from sample collection to disease diagnosis and asterisks indicating a confirmed postmortem AD diagnosis at autopsy. In the Venn diagram, ↑ arrows denote transcripts that are upregulated relative to the control group, while ↓ arrows mark downregulated transcripts. Significance is denoted in the box plots as ** (*p* < 0.01), and n.s. (not significant). The waterfall plot across the top of the heatmap demonstrates ELNET model confidence in assigning groups (0 to 1, with 0.5 being no confidence in the assignment), with dots colored to reference the diagnosis group.

AD exists on a continuum, where pathology precedes symptom onset sometimes by decades [55,56]. Disease-modifying therapy and interventions likely work best in the preclinical stage of the disease, making biomarkers that accurately diagnose AD early and predict disease progression valuable [57]. A subset of our control group (*n* = 6) converted to AD post-sample collection (Figure 2a). We compared the control to AD converter group (C to AD) with controls that did not change diagnosis and identified 384 significant transcripts, 63% of which were made up of miRNAs (Figure 4a). Strikingly, among the C to AD upregulated transcripts with the most significant *p*-values (*p* < 0.0001) were miR-125a, -191, and -23a, which were upregulated in AD (Figure 4b). Examining all significant DE transcripts in the converter group (relative to control, *p* < 0.05) and AD (relative to control, *p* < 0.05) showed 130 overlapping sequences, 98% of them concordant (Figure 4c). However, two thirds of the DE transcripts are unique to the converters, potentially representing a distinct early preclinical signature. For example, miR-494, which may be involved in neuroinflammation and microglial polarization, is exclusively upregulated in the C to AD group [58,59].

The number of years elapsed between the blood draw and the AD diagnosis in our converter group varied from two to twelve years. We examined whether expression levels of any transcripts correlate significantly with duration to conversion, and found over 70 genes with R^2^ = 0.66 or greater (*p* < 0.05) (Supplementary Materials Table S7). IsomiRs of miR-17 and -320a were downregulated as the manifestation of the disease approached and were likewise differentially expressed between the control and AD comparisons (Figure 4d). Finally, we tested whether our ELNET model between the control and AD could predict future disease, and found that five out of the six converters were identified as AD (Figure 4e).

**Figure 5:**
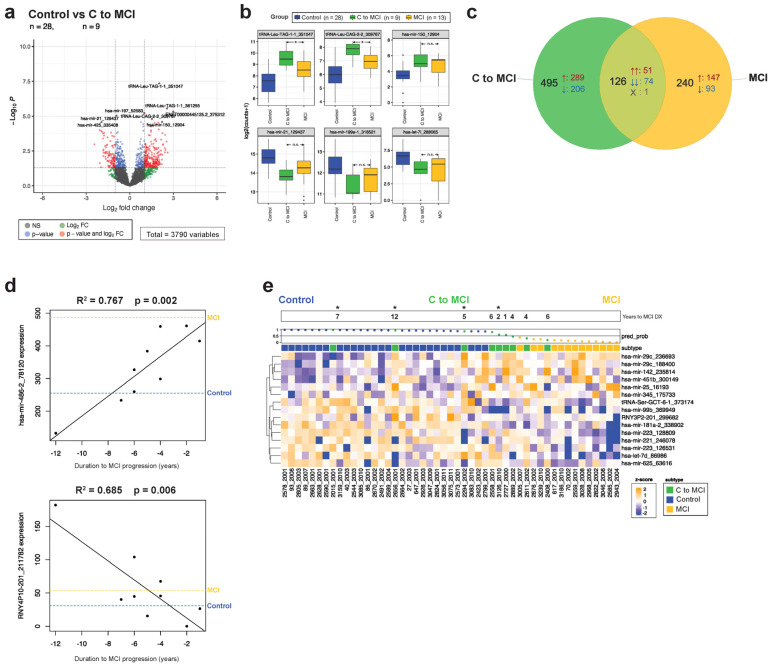
Converters to MCI express MCI-associated transcripts before diagnosis. (**a**) A total of 621 DE transcripts are significant when comparing the C to MCI group with the control group. (**b**) Box plots of representative significantly upregulated and downregulated sequences are displayed for the control, C to MCI, and MCI groups. (**c**) A Venn diagram comparing significant transcripts in each group relative to the control group shows 100 are overlapping, with the majority concordant. (**d**) Regression analysis plots by time to disease conversion with corresponding R^2^ and *p*-values are displayed, where the *X* axis of years to disease diagnosis is shown; the median expression level for the transcript in control non-converters (blue) and MCI (yellow) is provided as a reference. (**e**) A third of the MCI converters were identified as MCI by the ELNET model, while another third cluster near the midpoint of the model. In the Venn diagram, ↑ arrows denote transcripts that are upregulated relative to the control group, while ↓ arrows mark downregulated transcripts. Significance is denoted in the box plots as * (*p* < 0.05), and n.s. (not significant). The waterfall plot across the top of the heatmap demonstrates ELNET model confidence in assigning groups (0 to 1, with 0.5 being no confidence in the assignment), with dots colored to reference the diagnosis group. Asterisks across the waterfall plot indicate C to MCI converters with a confirmed AD diagnosis at autopsy.

A total of nine people originally classified as control converted to MCI (C to MCI) after sample collection, over the average span of 5.2 years (Figure 2a). Comparing them with controls that remained controls throughout the study revealed over 600 differentially expressed transcripts, some of which were significantly different in MCI as well (Figure 5a,b). Several isoforms of miR-320c, for example, which are exclusively upregulated in MCI (but not in AD or in C to AD), are likewise upregulated in the C to MCI group. Similar to the AD converters, the MCI converters also share 29% of their differential transcripts with MCI (Figure 5c). Regression analysis by time to conversion found over 260 transcripts (*p* < 0.05, Supplementary Materials Table S7). Mir-486-2 is upregulated with approaching MCI diagnosis and is differentially expressed between the control and MCI groups (Figure 5d). However, many transcripts that are correlated to disease progression are not significantly different between the static groups. miR-140, for example, was highly correlated with duration to diagnosis (R^2^ = 0.91), but not differentially expressed between the control and MCI groups (Supplementary Materials, Table S7).

The ELNET model distinguishing between control and MCI identified only a third of the converters as MCI, although several of the remaining converters clustered near the midpoint of the model (Figure 5e). Interestingly, the control vs. AD model was better than the MCI model at predicting disease in our MCI converters, as it identified seven out of nine participants in the disease group, particularly ones with confirmed AD pathology (Supplementary Materials Figure S2). Taken together, these data hint at the existence of an early, disease-specific EV signature in plasma that predates symptom onset by as much as a decade.

### 3.5. The EV-RNA Signature in MCI to AD Converters Foreshadows Disease Progression

**Figure 6:**
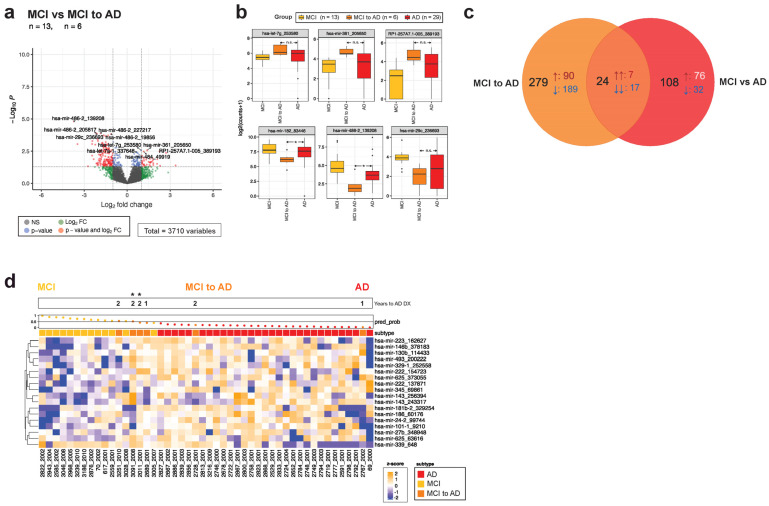
MCI participants who progressed to AD are accurately identified by their gene expression signature. (**a**) Comparing the MCI to AD converters to the MCI group that did not convert revealed 132 significantly differentially expressed transcripts. (**b**) Box plots show expression levels for representative DE sequences in the MCI, MCI to AD, and AD groups. (**c**) Overlap between significantly different transcripts in MCI to AD (relative to MCI) and AD (relative to MCI) is restricted to 15 genes as shown in a Venn diagram. (**d**) The ELNET model distinguishing AD from MCI identified most converters as AD. In the Venn diagram, ↑ arrows denote transcripts that are upregulated relative to the control group, while ↓ arrows mark downregulated transcripts. Significance is denoted in the box plots as * (*p* < 0.05), and n.s. (not significant). The waterfall plot across the top of the heatmap demonstrates ELNET model confidence in assigning groups (0 to 1, with 0.5 being no confidence in assignment), with dots colored to reference the diagnosis group. Asterisks across the waterfall plot indicate MCI to AD converters with a confirmed AD diagnosis at autopsy.

MCI is characterized by subtle cognitive change, particularly in memory, and positive brain pathology. While it is often thought of as an intermediate to AD, not all MCI cases become AD and duration to progression is variable, with some patients rapidly declining in months, whereas others may take over a decade to convert [60,61]. Therefore, identifying plasma RNA changes that can inform conversion status would be helpful in a clinical setting. A third of our MCI group converted to AD within 1–2 years (*n* = 6, MCI to AD), and differential gene expression between MCI and MCI converters identified 132 differentially expressed transcripts (Figure 6a). Expression of miR-let-7g, -361, and -29c isomiRs in converters was more similar to AD than to MCI, and all overlapping transcripts between MCI converters and AD were concordant (Figure 6b,c). Additionally, the AD vs. MCI ELNET model correctly identified four out of six converters as AD (Figure 6d).

Finally, numerous GWAS studies have described dozens of genetic variants strongly associated with AD risk, such as the ApoE locus, where ApoE allele 2 may have a protective effect or ApoE4 may have a detrimental role in neurodegeneration [62,63,64,65]. We examined ApoE genotyping for our cohort and found ApoE3/4 and 4/4 tended to be higher in our AD cohort, whereas ApoE2/3 status was higher in our control group (Supplementary Materials Table S1). Additionally, converters were more likely to be ApoE3/4 than their non-converter counterparts.

## 4. Discussion

Currently, minimally invasive and cost-effective biomarkers for diagnosing and monitoring AD progression that track clinical diagnosis/symptoms are needed. Many groups have attempted to quantify cell-free nucleic acid contents in human biofluids to achieve this goal, but considerable variability exists between studies, likely due to biofluid choice (plasma, serum, or CSF), isolation methods (extracellular vesicles vs. total cell-free RNA), experimental technique design (qRT-PCR vs. sequencing), and the cohorts chosen. In our study, we used plasma, which is more readily accessible and less invasive than CSF.

The choice of EV isolation method is a known source of variability among studies. It is difficult for findings to be consistent across isolation strategies, as each method provides unique advantages and disadvantages with respect to purity and yield. In this study, we used the exoRNeasy kit from Qiagen, an affinity-based EV capture method that can be used efficiently for larger numbers of samples and provides more uniform results across studies [37,38,66]. As indicated, this kit provided us with an opportunity to look at small RNAs associated with EVs and an EV-depleted fraction. We compared the small RNA cargo in EVs with the EV-depleted fraction associated with circulating protein complexes, like HDL, LDL, or RNPs. Regardless of the experimental group, EV-RNA was significantly less variable and more likely to contain unique small RNA transcripts not found in the EV-depleted portion (Figure 1).

AD is a complex neurodegenerative condition often diagnosed long after molecular and pathological changes have begun. Early in the disease, AD is more difficult to separate from dementias caused by other pathologies or vascular changes, which complicates biomarker research and further contributes to variability. Here, we leveraged the unique longitudinal care of our cohort and postmortem confirmation of several participants. This allowed us to exclude patients with a clinical diagnosis that differed from autopsy reports and to create subsets of “preclinical” converters that allowed us to study disease-associated changes that precede cognitive symptoms (Figure 2).

Differential gene expression between the control, MCI, and AD groups found hundreds of significantly different sequences, spanning all small RNA biotypes (Figure 3). Several isomiRs of miR-125a, which may modulate glial function and have been previously described as upregulated in AD and MS, are upregulated in our cohort [67,68,69]. miRNAs associated with neuroinflammation, like miR-142 and -146a, are differentially regulated in our AD group [70,71,72,73]. While MCI shares a portion of its differentially expressed genes with AD, almost two thirds of its differential transcripts are unique, suggesting a distinct, MCI-specific molecular signature. This is noteworthy, as not all MCI cases progress to AD. Long-term, longitudinal studies exploring plasma EV-RNA in individuals diagnosed with MCI may shed light on predictive biomarkers for disease trajectory. ELNET regression modeling based on differential transcripts can distinguish disease status with 78–83% accuracy, using 15–23 transcripts predominantly made up of miRNAs. Larger scale data sets and combinations with other biomarkers may improve accuracy and AUC in future studies.

A number of patients with no diagnosed cognitive impairments at the time of sample collection received an MCI or AD diagnosis 2–12 years later. Remarkably, expression levels of certain disease-associated miRNAs, like miR-125a, -142, -191, -17, and -150 in the converter groups, were significantly different compared with the controls and instead were more similar to expression levels observed in the AD and MCI groups (Figure 4 and Figure 5). Other small RNA species, particularly YRNA and tRNAs, followed the same trend. Furthermore, several transcripts were significantly associated with the duration of time elapsed to AD or MCI diagnosis, although a large portion of the differential genes in the converter groups were unique, and not shared with either disease. Trying to predict conversion using ELNET yielded promising initial results, particularly in the AD converters, where all individuals who developed AD within a decade of the sample collection were correctly identified by the model (Figure 4). This may be further refined by incorporating the early converter-specific EV-RNA signature into the model as well. Predicting MCI conversion with the MCI model was less successful, with only three out of nine being identified as MCI, possibly reflecting the heterogeneous nature of an MCI diagnosis (Figure 5). Finally, a third of our MCI group converted to AD shortly after sample collection, and the ELNET model was able to predict conversion in four out of six participants (Figure 6).

We recognize our sample size, particularly for the converter subsets, is small (C to MCI, *n* = 9; C to AD, *n* = 6; and MCI to AD, *n* = 6). In addition, the clinical characterization of the cohort available to us included DRS and MMSE scores, and postmortem pathology information for only a subset of participants. Larger scale studies with subjects who are followed longitudinally and converted from control to MCI or AD are needed to confirm and expand upon our findings. Ideally, studies that have the ability to span decades and consider both physician-provided diagnoses and molecular pathological findings will be crucial for identifying biomarkers with robust clinical utility. Together, our data highlight the importance of integrating clinical information when defining experimental groups and suggest that quantifiable and significant disease-associated changes are present in the plasma EVs of pre-clinical patients years before symptom onset. These changes could be leveraged to predict disease and monitor progression in a clinical setting.

## Data Availability

All aggregated sequencing data, including counts for individual RNAs, can be found in the ERCC extracellular RNA atlas, accession EXR-KJENS1yrpQwk-AN (https://exrna-atlas.org/exat/datasets/) or in SRA, accession PRJNA972172 ID: 972172.

## Supplementary Materials

The following supporting information can be downloaded at TBD https://www.mdpi.com/article/10.3390/cells13030207/s1, Figure S1: ELNET modeling to distinguish between different groups based on post mortem pathology findings; Figure S2: ELNET model distinguishing control and AD participants predicts conversion in the control groups; Table S1: Cohort metadata and sample information; Table S2: Gene ID look-up table; Table S3: Read fractions for EV enriched and EV depleted samples; Table S4: DEG analysis; Table S5: Compiled ELNET metrics; Table S6: Compiled LRM metrics; Table S7: Linear regression analysis.
