# Supplementary figures and images for: Small RNA Changes in Plasma Have Potential for Early Diagnosis of Alzheimer’s Disease before Symptom Onset
Source: Cells. 2024 Jan 23;13(3):207. doi: 10.3390/cells13030207 (PMC10854972; doi:10.3390/cells13030207)

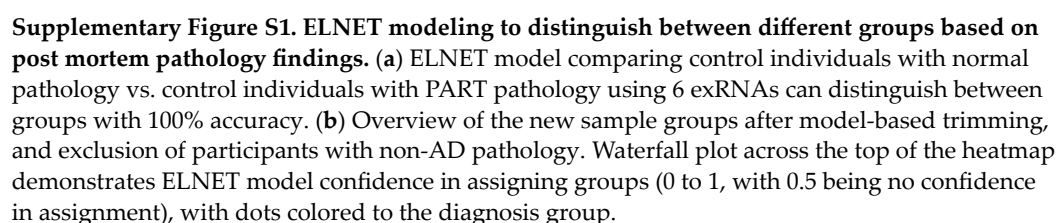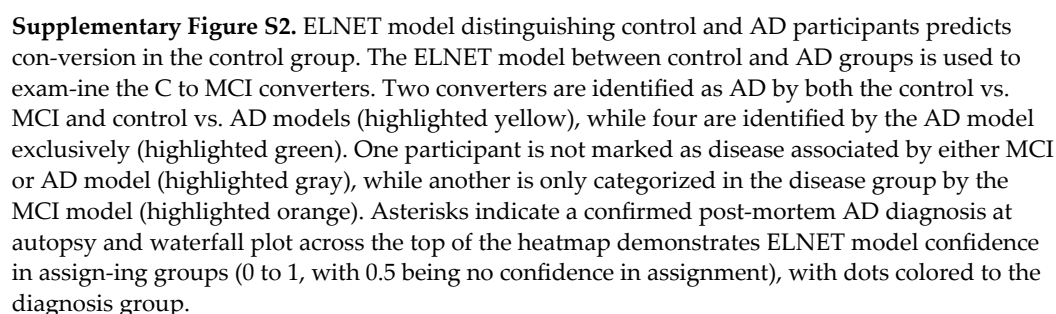

Supplement: Supplementary file 1 [file cells-13-00207-s001.zip › supplementary figures.pdf]
